# Dieth­yl[*N*-(3-meth­oxy-2-oxidobenzyl­idene)-*N*′-(oxidomethyl­ene)hydrazine-κ^3^ *O*,*N*,*O*′]tin(IV)

**DOI:** 10.1107/S1600536808018953

**Published:** 2008-06-28

**Authors:** Shaukat Shuja, M. Nawaz Tahir, Saqib Ali, Nasir Khalid

**Affiliations:** a Department of Chemistry, Quaid-i-Azam University, Islamabad 45320, Pakistan; b University of Sargodha, Department of Physics, Sagrodha, Pakistan; c Chemistry Division, Pakistan Institute of Nuclear Science and Technology, PO Nilore, Islamabad, Pakistan

## Abstract

In the mol­ecule of the title compound, [Sn(C_2_H_5_)_2_(C_9_H_8_N_2_O_3_)], the Sn atom is five-coordinated in a distorted trigonal-bipyramidal configuration by two O and one N atoms of the tridentate Schiff base ligand in the equatorial plane, and by two C atoms of ethyl groups in the axial positions. In the crystal structure, inter­molecular C—H⋯O hydrogen bonds link the mol­ecules into centrosymmetric dimers.

## Related literature

For related literature, see: Chen *et al.* (2006 ▶); Shuja *et al.* (2007*a* ▶,*b* ▶,*c* ▶); Shuja *et al.* (2008 ▶). For ring puckering parameters, see: Cremer & Pople (1975 ▶). 
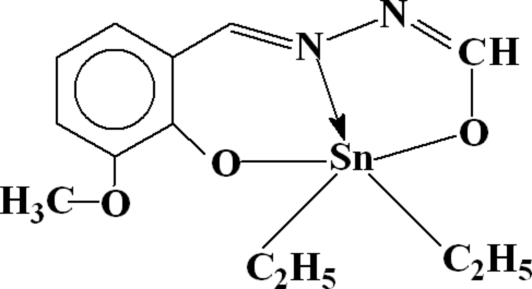


## Experimental

### 

#### Crystal data

[Sn(C_2_H_5_)_2_(C_9_H_8_N_2_O_3_)]
*M* *_r_* = 368.98
Triclinic, 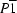

*a* = 8.2485 (3) Å
*b* = 9.8609 (4) Å
*c* = 10.4501 (4) Å
α = 63.521 (2)°
β = 68.967 (1)°
γ = 77.803 (2)°
*V* = 708.79 (5) Å^3^
*Z* = 2
Mo *K*α radiation
μ = 1.81 mm^−1^
*T* = 296 (2) K
0.30 × 0.20 × 0.18 mm

#### Data collection

Bruker Kappa APEXII CCD diffractometer
Absorption correction: multi-scan (*SADABS*; Bruker, 2005 ▶) *T*_min_ = 0.650, *T*_max_ = 0.720
14275 measured reflections
3603 independent reflections
3458 reflections with *I* > 2σ(*I*)
*R*_int_ = 0.023

#### Refinement

*R*[*F* ^2^ > 2σ(*F* ^2^)] = 0.016
*wR*(*F* ^2^) = 0.063
*S* = 1.02
3603 reflections
226 parameters
H-atom parameters constrained
Δρ_max_ = 0.39 e Å^−3^
Δρ_min_ = −0.59 e Å^−3^

### 

Data collection: *APEX2* (Bruker, 2007 ▶); cell refinement: *APEX2*; data reduction: *SAINT* (Bruker, 2007 ▶); program(s) used to solve structure: *SHELXS97* (Sheldrick, 2008 ▶); program(s) used to refine structure: *SHELXL97* (Sheldrick, 2008 ▶); molecular graphics: *ORTEP-3 for Windows* (Farrugia, 1997 ▶); software used to prepare material for publication: *WinGX* (Farrugia, 1999 ▶) and *PLATON* (Spek, 2003 ▶).

## Supplementary Material

Crystal structure: contains datablocks global, I. DOI: 10.1107/S1600536808018953/hk2477sup1.cif

Structure factors: contains datablocks I. DOI: 10.1107/S1600536808018953/hk2477Isup2.hkl

Additional supplementary materials:  crystallographic information; 3D view; checkCIF report

## Figures and Tables

**Table:** 

Sn1—C11	2.1216 (19)
Sn1—C9	2.1217 (18)
Sn1—O1	2.1888 (13)
Sn1—O2	2.2162 (14)
Sn1—N1	2.2271 (15)

**Table:** 

C11—Sn1—C9	153.45 (9)
C11—Sn1—O1	97.19 (7)
C9—Sn1—O1	93.40 (7)
C11—Sn1—O2	92.71 (8)
C9—Sn1—O2	88.78 (7)
O1—Sn1—O2	152.79 (5)
C11—Sn1—N1	99.42 (7)
C9—Sn1—N1	106.13 (7)
O1—Sn1—N1	82.07 (5)
O2—Sn1—N1	71.30 (6)

**Table 2:** Hydrogen-bond geometry (Å, °)

*D*—H⋯*A*	*D*—H	H⋯*A*	*D*⋯*A*	*D*—H⋯*A*
C13—H13*A*⋯O2^i^	0.96	2.34	3.068 (4)	132

Symmetry code: (i) 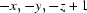.

**Fig. 1.**
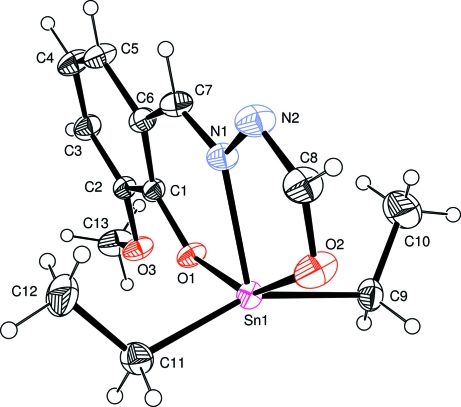
The molecular structure of the title molecule, with the atom-numbering scheme. Displacement ellipsoids are drawn at the 30% probability level.

**Fig. 2.**
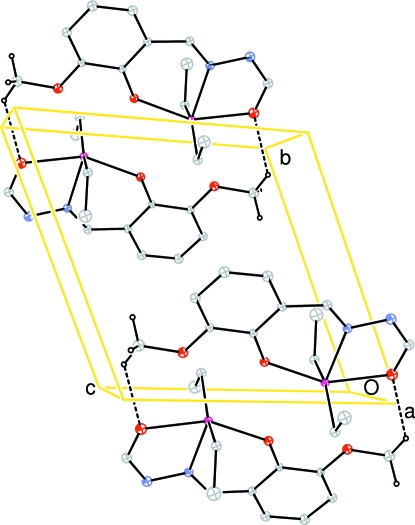
A partial packing diagram of (I). Hydrogen bonds are shown as dashed lines.

## supplementary crystallographic information

### Comment

In continuation of our efforts to synthesize various Schiff base ligands of substituted salicylaldehydes with hydrazides, aminoacids and their organotin derivatives (Shuja *et al.*,  2007*a*,b,c; Shuja *et al.*,  2008), we report herein the crystal structure of the title compound, (I).

In the molecule of (I) (Fig. 1), the Sn atom is five-coordinated in distorted trigonal bipyramidal configuration (Table 1) by two O and one N atoms of the tridentate Schiff base ligand in the equatorial plane, and two C atoms of diethyl groups in the axial positions. The bond lengths and angles are within normal ranges, which are comparable with the corresponding values in (3-methoxy-2-oxidobenzaldehyde benzoylhydrazonato)dimethyltin(IV), (II) (Chen *et al.*,  2006) and (2,2'-bipyridine-κ^2^N,N'){[(3-methoxy-2-oxidobenzylidene -κO^2^)hydrazono]methanolato-κ^2^N^2^,O}dimethyltin(IV), (III) (Shuja *et al.*,  2008). The Sn1-C9 [2.1217 (18) Å] and Sn1-C11 [2.1219 (19) Å] bonds in (I) are reported as 2.099 (4) and 2.102 (4) Å in (II) and 2.097 (3) and 2.098 (3) Å in (III). On the other hand, the Sn1-O1 [2.1888 (13) Å] and Sn1-O2 [2.2162 (14) Å] bonds in (I) are reported as 2.131 (3) and 2.178 (3) Å in (II) and 2.1572 (14) and 2.2658 (15) Å in (III), while the Sn1-N1 [2.2271 (15) Å] bond in (I) is reported as 2.188 (3) Å in (II) and 2.2980 (18) Å in (III).

Rings A (Sn1/O2/N1/N2/C8) and C (C1-C6) are, of course, planar, and the dihedral angle between them is A/C = 7.96 (3)°. Ring B (Sn1/O1/N1/C1/C6/C7) adopts flattened-boat [φ = -57.24 (2)° and θ = 107.39 (3)°] conformation, having total puckering amplitude, Q_T_, of 0.453 (3) Å (Cremer & Pople, 1975).

In the crystal structure, intermolecular C-H···O hydrogen bonds (Table 2) link the molecules into centrosymmetric dimers (Fig. 2), in which they may be effective in the stabilization of the structure.

### Experimental

For the preparation of the title compound, N-(3-methoxy-2-hydroxybenzylidene)- formichydrazide (0.58 g, 3.0 mmol) and Et_3_N (0.84 ml, 6 mmol) were added to dry toluene (100 ml) in a 250 ml round bottom flask equipped with a reflux condenser. Diethyltin(IV) dichloride (0.74 g, 3.0 mmol) dissolved in dry toluene (20 ml) was then added. The reaction mixture was stirred at room temperature for 5 h and allowed to stand overnight. The formed Et_3_N-HCl was filtered off and the clear yellow solution was evaporated on a rotary evaporator under reduced pressure. Crystals of (I) were obtained by recrystallization from a chloroform solution.

### Crystal data

**Table:**

[Sn(C_2_H_5_)_2_(C_9_H_8_N_2_O_3_)]	*Z* = 2
*M**_r_* = 368.98	*F*_000_ = 368
Triclinic, *P*1	*D*_x_ = 1.729 Mg m^−^^3^
Hall symbol: -P 1	Mo *K*α radiation λ = 0.71073 Å
*a* = 8.2485 (3) Å	Cell parameters from 3603 reflections
*b* = 9.8609 (4) Å	θ = 2.3–28.7º
*c* = 10.4501 (4) Å	µ = 1.81 mm^−^^1^
α = 63.521 (2)º	*T* = 296 (2) K
β = 68.967 (1)º	Prismatic, yellow
γ = 77.803 (2)º	0.30 × 0.20 × 0.18 mm
*V* = 708.79 (5) Å^3^	

### Data collection

**Table:**

Bruker Kappa APEXII CCD diffractometer	3603 independent reflections
Radiation source: fine-focus sealed tube	3458 reflections with *I* > 2σ(*I*)
Monochromator: graphite	*R*_int_ = 0.023
Detector resolution: 7.4 pixels mm^-1^	θ_max_ = 28.7º
*T* = 296(2) K	θ_min_ = 2.3º
ω scans	*h* = −11→11
Absorption correction: multi-scan(SADABS; Bruker, 2005)	*k* = −13→13
*T*_min_ = 0.650, *T*_max_ = 0.720	*l* = −14→14
14275 measured reflections	

### Refinement

**Table:**

Refinement on *F*^2^	Secondary atom site location: difference Fourier map
Least-squares matrix: full	Hydrogen site location: inferred from neighbouring sites
*R*[*F*^2^ > 2σ(*F*^2^)] = 0.016	H-atom parameters constrained
*wR*(*F*^2^) = 0.063	*w* = 1/[σ^2^(*F*_o_^2^) + (0.0503*P*)^2^ + 0.0901*P*] where *P* = (*F*_o_^2^ + 2*F*_c_^2^)/3
*S* = 1.02	(Δ/σ)_max_ = 0.001
3603 reflections	Δρ_max_ = 0.39 e Å^−^^3^
226 parameters	Δρ_min_ = −0.59 e Å^−^^3^
Primary atom site location: structure-invariant direct methods	Extinction correction: none

### Special details

**Table:**

Geometry. All e.s.d.'s (except the e.s.d. in the dihedral angle between two l.s. planes) are estimated using the full covariance matrix. The cell e.s.d.'s are taken into account individually in the estimation of e.s.d.'s in distances, angles and torsion angles; correlations between e.s.d.'s in cell parameters are only used when they are defined by crystal symmetry. An approximate (isotropic) treatment of cell e.s.d.'s is used for estimating e.s.d.'s involving l.s. planes.
Refinement. Refinement of F^2^ against ALL reflections. The weighted R-factor wR and goodness of fit S are based on F^2^, conventional R-factors R are based on F, with F set to zero for negative F^2^. The threshold expression of F^2^ > 2sigma(F^2^) is used only for calculating R-factors(gt) etc. and is not relevant to the choice of reflections for refinement. R-factors based on F^2^ are statistically about twice as large as those based on F, and R- factors based on ALL data will be even larger.

### Fractional atomic coordinates and isotropic or equivalent isotropic displacement parameters (Å^2^)

**Table:**

	*x*	*y*	*z*	*U*_iso_*/*U*_eq_	
Sn1	0.134568 (12)	0.080659 (11)	0.280902 (10)	0.02876 (6)	
O1	0.07311 (18)	0.14980 (15)	0.46603 (15)	0.0333 (3)	
O2	0.2427 (2)	0.11910 (19)	0.04108 (16)	0.0510 (4)	
O3	−0.04789 (19)	0.17238 (16)	0.73028 (15)	0.0400 (3)	
N1	0.2910 (2)	0.28451 (17)	0.15785 (16)	0.0343 (3)	
N2	0.3750 (3)	0.3247 (2)	0.0026 (2)	0.0446 (4)	
C1	0.1382 (2)	0.25342 (18)	0.47969 (19)	0.0276 (3)	
C2	0.0784 (2)	0.26822 (19)	0.6190 (2)	0.0303 (3)	
C3	0.1440 (3)	0.3722 (2)	0.6396 (2)	0.0396 (4)	
H3	0.1024	0.3790	0.7320	0.048*	
C4	0.2714 (3)	0.4670 (3)	0.5238 (3)	0.0478 (5)	
H4	0.3166	0.5354	0.5392	0.057*	
C5	0.3298 (3)	0.4587 (2)	0.3872 (3)	0.0447 (4)	
H5	0.4144	0.5225	0.3095	0.054*	
C6	0.2634 (2)	0.3545 (2)	0.3624 (2)	0.0334 (3)	
C7	0.3300 (3)	0.3656 (2)	0.2102 (2)	0.0365 (4)	
H7	0.4095	0.4390	0.1427	0.044*	
C8	0.3378 (3)	0.2327 (3)	−0.0403 (2)	0.0443 (4)	
H8	0.3857	0.2517	−0.1416	0.053*	
C9	−0.1144 (2)	0.1569 (2)	0.2494 (2)	0.0394 (4)	
H9A	−0.1301	0.1149	0.1869	0.047*	
H9B	−0.2029	0.1188	0.3460	0.047*	
C10	−0.1400 (4)	0.3268 (3)	0.1781 (5)	0.0777 (9)	
H10A	−0.2545	0.3544	0.1668	0.116*	
H10B	−0.0547	0.3653	0.0813	0.116*	
H10C	−0.1274	0.3692	0.2405	0.116*	
C11	0.3323 (3)	−0.0893 (2)	0.3408 (2)	0.0413 (4)	
H11A	0.2880	−0.1564	0.4457	0.050*	
H11B	0.3580	−0.1491	0.2824	0.050*	
C12	0.4986 (3)	−0.0295 (3)	0.3172 (4)	0.0713 (8)	
H12A	0.5810	−0.1129	0.3468	0.107*	
H12B	0.4755	0.0276	0.3767	0.107*	
H12C	0.5456	0.0350	0.2132	0.107*	
C13	−0.1177 (4)	0.1920 (3)	0.8682 (2)	0.0545 (6)	
H13A	−0.2039	0.1197	0.9374	0.082*	
H13B	−0.1702	0.2930	0.8493	0.082*	
H13C	−0.0259	0.1764	0.9103	0.082*	

### Atomic displacement parameters (Å^2^)

**Table:**

	*U*^11^	*U*^22^	*U*^33^	*U*^12^	*U*^13^	*U*^23^
Sn1	0.02673 (8)	0.03389 (8)	0.02623 (8)	−0.00313 (5)	−0.00603 (5)	−0.01366 (6)
O1	0.0369 (7)	0.0368 (6)	0.0307 (6)	−0.0122 (5)	−0.0037 (5)	−0.0182 (5)
O2	0.0609 (10)	0.0622 (10)	0.0326 (7)	−0.0265 (8)	0.0021 (6)	−0.0241 (7)
O3	0.0483 (8)	0.0442 (7)	0.0324 (6)	−0.0180 (6)	−0.0017 (6)	−0.0213 (6)
N1	0.0332 (7)	0.0351 (7)	0.0284 (7)	−0.0083 (6)	−0.0023 (6)	−0.0103 (6)
N2	0.0493 (10)	0.0467 (9)	0.0286 (8)	−0.0174 (8)	0.0030 (7)	−0.0126 (7)
C1	0.0275 (7)	0.0272 (7)	0.0323 (7)	−0.0016 (6)	−0.0111 (6)	−0.0139 (6)
C2	0.0313 (8)	0.0292 (8)	0.0339 (8)	−0.0025 (6)	−0.0118 (7)	−0.0140 (7)
C3	0.0465 (10)	0.0410 (9)	0.0420 (9)	−0.0065 (8)	−0.0148 (8)	−0.0233 (8)
C4	0.0537 (12)	0.0470 (11)	0.0559 (12)	−0.0178 (9)	−0.0141 (10)	−0.0276 (10)
C5	0.0468 (11)	0.0409 (10)	0.0481 (11)	−0.0190 (8)	−0.0081 (9)	−0.0172 (9)
C6	0.0321 (8)	0.0319 (8)	0.0376 (8)	−0.0059 (6)	−0.0095 (7)	−0.0143 (7)
C7	0.0354 (9)	0.0332 (8)	0.0356 (9)	−0.0105 (7)	−0.0023 (7)	−0.0123 (7)
C8	0.0459 (10)	0.0511 (11)	0.0283 (8)	−0.0121 (9)	0.0007 (7)	−0.0149 (8)
C9	0.0319 (8)	0.0483 (10)	0.0361 (9)	−0.0043 (7)	−0.0132 (7)	−0.0124 (8)
C10	0.0660 (17)	0.0505 (14)	0.112 (3)	0.0168 (13)	−0.0459 (18)	−0.0229 (16)
C11	0.0344 (9)	0.0382 (9)	0.0488 (10)	0.0038 (7)	−0.0101 (8)	−0.0200 (8)
C12	0.0440 (13)	0.0669 (16)	0.118 (3)	0.0075 (11)	−0.0433 (15)	−0.0406 (17)
C13	0.0744 (16)	0.0556 (12)	0.0370 (10)	−0.0203 (11)	−0.0005 (10)	−0.0274 (10)

### Geometric parameters (Å, °)

**Table:**

Sn1—C11	2.1216 (19)	C5—H5	0.9300
Sn1—C9	2.1217 (18)	C6—C7	1.444 (3)
Sn1—O1	2.1888 (13)	C7—H7	0.9300
Sn1—O2	2.2162 (14)	C8—H8	0.9300
Sn1—N1	2.2271 (15)	C9—C10	1.502 (3)
O1—C1	1.3304 (19)	C9—H9A	0.9700
O2—C8	1.278 (3)	C9—H9B	0.9700
O3—C2	1.377 (2)	C10—H10A	0.9600
O3—C13	1.433 (2)	C10—H10B	0.9600
N1—C7	1.291 (2)	C10—H10C	0.9600
N1—N2	1.416 (2)	C11—C12	1.504 (3)
N2—C8	1.304 (3)	C11—H11A	0.9700
C1—C6	1.414 (2)	C11—H11B	0.9700
C1—C2	1.424 (2)	C12—H12A	0.9600
C2—C3	1.380 (2)	C12—H12B	0.9600
C3—C4	1.392 (3)	C12—H12C	0.9600
C3—H3	0.9300	C13—H13A	0.9600
C4—C5	1.368 (3)	C13—H13B	0.9600
C4—H4	0.9300	C13—H13C	0.9600
C5—C6	1.414 (2)		
			
C11—Sn1—C9	153.45 (9)	N1—C7—H7	116.5
C11—Sn1—O1	97.19 (7)	C6—C7—H7	116.5
C9—Sn1—O1	93.40 (7)	O2—C8—N2	127.32 (18)
C11—Sn1—O2	92.71 (8)	O2—C8—H8	116.3
C9—Sn1—O2	88.78 (7)	N2—C8—H8	116.3
O1—Sn1—O2	152.79 (5)	C10—C9—Sn1	113.26 (16)
C11—Sn1—N1	99.42 (7)	C10—C9—H9A	108.9
C9—Sn1—N1	106.13 (7)	Sn1—C9—H9A	108.9
O1—Sn1—N1	82.07 (5)	C10—C9—H9B	108.9
O2—Sn1—N1	71.30 (6)	Sn1—C9—H9B	108.9
C1—O1—Sn1	131.78 (11)	H9A—C9—H9B	107.7
C8—O2—Sn1	114.11 (13)	C9—C10—H10A	109.5
C2—O3—C13	116.47 (15)	C9—C10—H10B	109.5
C7—N1—N2	113.77 (15)	H10A—C10—H10B	109.5
C7—N1—Sn1	129.00 (12)	C9—C10—H10C	109.5
N2—N1—Sn1	116.91 (12)	H10A—C10—H10C	109.5
C8—N2—N1	110.32 (16)	H10B—C10—H10C	109.5
O1—C1—C6	124.13 (15)	C12—C11—Sn1	114.55 (16)
O1—C1—C2	119.40 (15)	C12—C11—H11A	108.6
C6—C1—C2	116.45 (15)	Sn1—C11—H11A	108.6
O3—C2—C3	122.78 (16)	C12—C11—H11B	108.6
O3—C2—C1	115.59 (14)	Sn1—C11—H11B	108.6
C3—C2—C1	121.62 (17)	H11A—C11—H11B	107.6
C2—C3—C4	120.80 (18)	C11—C12—H12A	109.5
C2—C3—H3	119.6	C11—C12—H12B	109.5
C4—C3—H3	119.6	H12A—C12—H12B	109.5
C5—C4—C3	119.43 (17)	C11—C12—H12C	109.5
C5—C4—H4	120.3	H12A—C12—H12C	109.5
C3—C4—H4	120.3	H12B—C12—H12C	109.5
C4—C5—C6	120.97 (19)	O3—C13—H13A	109.5
C4—C5—H5	119.5	O3—C13—H13B	109.5
C6—C5—H5	119.5	H13A—C13—H13B	109.5
C1—C6—C5	120.65 (17)	O3—C13—H13C	109.5
C1—C6—C7	124.88 (16)	H13A—C13—H13C	109.5
C5—C6—C7	114.44 (17)	H13B—C13—H13C	109.5
N1—C7—C6	126.99 (17)		
			
C11—Sn1—O1—C1	89.09 (16)	C6—C1—C2—C3	−2.2 (3)
C9—Sn1—O1—C1	−115.31 (16)	O3—C2—C3—C4	−179.90 (19)
O2—Sn1—O1—C1	−21.4 (2)	C1—C2—C3—C4	0.1 (3)
N1—Sn1—O1—C1	−9.48 (15)	C2—C3—C4—C5	1.3 (3)
C11—Sn1—O2—C8	−97.99 (17)	C3—C4—C5—C6	−0.5 (4)
C9—Sn1—O2—C8	108.54 (17)	O1—C1—C6—C5	−178.21 (17)
O1—Sn1—O2—C8	13.5 (3)	C2—C1—C6—C5	3.0 (3)
N1—Sn1—O2—C8	1.05 (16)	O1—C1—C6—C7	3.8 (3)
C11—Sn1—N1—C7	−85.14 (18)	C2—C1—C6—C7	−175.00 (18)
C9—Sn1—N1—C7	102.15 (18)	C4—C5—C6—C1	−1.7 (3)
O1—Sn1—N1—C7	10.88 (17)	C4—C5—C6—C7	176.4 (2)
O2—Sn1—N1—C7	−174.82 (19)	N2—N1—C7—C6	178.61 (19)
C11—Sn1—N1—N2	87.99 (15)	Sn1—N1—C7—C6	−8.1 (3)
C9—Sn1—N1—N2	−84.72 (15)	C1—C6—C7—N1	−1.9 (3)
O1—Sn1—N1—N2	−175.99 (16)	C5—C6—C7—N1	−180.0 (2)
O2—Sn1—N1—N2	−1.69 (14)	Sn1—O2—C8—N2	−0.3 (3)
C7—N1—N2—C8	176.23 (19)	N1—N2—C8—O2	−1.2 (3)
Sn1—N1—N2—C8	2.1 (2)	C11—Sn1—C9—C10	−173.4 (2)
Sn1—O1—C1—C6	4.8 (3)	O1—Sn1—C9—C10	73.0 (2)
Sn1—O1—C1—C2	−176.49 (12)	O2—Sn1—C9—C10	−79.8 (2)
C13—O3—C2—C3	3.9 (3)	N1—Sn1—C9—C10	−9.7 (2)
C13—O3—C2—C1	−176.17 (18)	C9—Sn1—C11—C12	176.3 (2)
O1—C1—C2—O3	−1.0 (2)	O1—Sn1—C11—C12	−71.0 (2)
C6—C1—C2—O3	177.81 (15)	O2—Sn1—C11—C12	83.6 (2)
O1—C1—C2—C3	178.91 (17)	N1—Sn1—C11—C12	12.1 (2)

### Hydrogen-bond geometry (Å, °)

**Table:**

*D*—H···*A*	*D*—H	H···*A*	*D*···*A*	*D*—H···*A*
C13—H13A···O2^i^	0.96	2.34	3.068 (4)	132

Symmetry codes: (i) −*x*, −*y*, −*z*+1.
